# Spatio-Temporal Expression Profile of Stem Cell-Associated Gene LGR5 in the Intestine during Thyroid Hormone-Dependent Metamorphosis in *Xenopus laevis*

**DOI:** 10.1371/journal.pone.0013605

**Published:** 2010-10-22

**Authors:** Guihong Sun, Takashi Hasebe, Kenta Fujimoto, Rosemary Lu, Liezhen Fu, Hiroki Matsuda, Mitsuko Kajita, Atsuko Ishizuya-Oka, Yun-Bo Shi

**Affiliations:** 1 Section on Molecular Morphogenesis, Laboratory of Gene Regulation and Development, Program in Cellular Regulation and Metabolism (PCRM), Eunice Kennedy Shriver National Institute of Child Health and Human Development (NICHD), National Institutes of Health (NIH), Bethesda, Maryland, United States of America; 2 Department of Biology, Nippon Medical School, Kawasaki, Kanagawa, Japan; 3 Department of Molecular Biology, Institute of Development and Aging Sciences, Nippon Medical School, Kawasaki, Kanagawa, Japan; University of Colorado, Boulder, United States of America

## Abstract

**Background:**

The intestinal epithelium undergoes constant self-renewal throughout adult life across vertebrates. This is accomplished through the proliferation and subsequent differentiation of the adult stem cells. This self-renewal system is established in the so-called postembryonic developmental period in mammals when endogenous thyroid hormone (T3) levels are high.

**Methodology/Principal Findings:**

The T3-dependent metamorphosis in anurans like *Xenopus laevis* resembles the mammalian postembryonic development and offers a unique opportunity to study how the adult stem cells are developed. The tadpole intestine is predominantly a monolayer of larval epithelial cells. During metamorphosis, the larval epithelial cells undergo apoptosis and, concurrently, adult epithelial stem/progenitor cells develop *de novo*, rapidly proliferate, and then differentiate to establish a trough-crest axis of the epithelial fold, resembling the crypt-villus axis in the adult mammalian intestine. The leucine-rich repeat-containing G protein-coupled receptor 5 (LGR5) is a well-established stem cell marker in the adult mouse intestinal crypt. Here we have cloned and analyzed the spatiotemporal expression profile of LGR5 gene during frog metamorphosis. We show that the two duplicated LGR5 genes in *Xenopus laevis* and the LGR5 gene in *Xenopus tropicalis* are highly homologous to the LGR5 in other vertebrates. The expression of LGR5 is induced in the limb, tail, and intestine by T3 during metamorphosis. More importantly, LGR5 mRNA is localized to the developing adult epithelial stem cells of the intestine.

**Conclusions/Significance:**

These results suggest that LGR5-expressing cells are the stem/progenitor cells of the adult intestine and that LGR5 plays a role in the development and/or maintenance of the adult intestinal stem cells during postembryonic development in vertebrates.

## Introduction

Adult organ-specific stem cells are critical for organ regeneration and repair. They are also essential for the physiological function of many adult organs that undergo self-renewal. One of the best studied such organs is the intestine. In the adult vertebrate intestine, the epithelium is responsible for the food digestion and absorption of nutrients and is constantly self-renewed [1], [2], [3], [4], [5]. This occurs through proliferation of the stem cells followed by their differentiation. The differentiated epithelial cells undergo cell death and are sloughed into the lumen after a finite period of time. Such an interesting property has prompted extensive studies, leading to the identification of a number of signaling pathways required for intestinal development and cell renewal in the adult [3], [6], [7]. On the other hand, how the adult stem cells are formed during development is largely unknown as it is difficult to manipulate and study mammalian embryos enclosed in the uterus.

Amphibian metamorphosis mimics the so-called post-embryonic development, a period from a few months before to several months after birth in human when plasma thyroid hormone (T3) concentrations are high [8]. During metamorphosis, essentially all organs/tissues are transformed as a tadpole is changed into a frog in a process entirely controlled by T3 [9], [10]. There are a number of similarities between anuran metamorphosis and postembryonic development in mammals, including the maturation of the intestine into the adult form. In *Xenopus laevis*, the tadpole intestine consists of mostly a monolayer of larval epithelial cells [5]. When T3 becomes available either naturally or after addition to the rearing water, metamorphosis is induced and the larval epithelial cells undergo apoptosis and concurrently, adult epithelial stem/progenitor cells rapidly proliferate [5], [11], [12]. By the end of metamorphosis, the epithelial cells differentiate to establish a trough-crest axis of epithelial fold, resembling the crypt-villus axis in the adult mammalian intestine [5]. Like metamorphosis itself, intestinal remodeling can be reproduced by T3-treatment of premetamorphic tadpoles or even in organ cultures of tadpole intestine *in vitro* [11], [13], [14].

The organ-autonomous induction of intestinal remodeling during metamorphosis makes a valuable system to study the development of the adult organ-specific stem cells. Immunohistochemical and recombinant organ culture studies have provided strong evidence that the progenitor/stem cells of the adult intestinal epithelium are derived from the larval epithelium in *Xenopus laevis* through yet unknown mechanism and rapidly proliferate [5], [11], [12], [15]. To further study the underlying molecular mechanism for the development and maintenance of these adult stem cells, we have cloned and characterized the orphan leucine-rich repeat G protein-coupled receptor 5 (LGR5), a well established stem cell marker in the adult mouse intestinal crypt [16], [17], [18]. LGR5 is a member of the glycoprotein hormone receptor family [19] but its ligand and physiological function is yet unclear. LGR5 gene knockout in mouse results in neonatal lethality due to a developmental defect in the tongue [20]. LGR5 deficiency causes precocious Paneth cell differentiation in the fetal intestine [21], suggesting that LGR5 regulates intestinal stem cell differentiation in mouse. Here we show that LGR5 is induced in the limb, tail, and intestine by T3 during metamorphosis and LGR5 expression is localized to the developing adult epithelial stem cells of the intestine. These results suggest that LGR5 plays a role in the development and/or maintenance of the intestinal stem cells during metamorphosis and also serves as a good molecular marker for future studies on postembryonic adult organ-specific stem cell development in vertebrates.

## Materials and Methods

### Experimental animals

All experiments involving *X. laevis* animals were conducted in accordance with accepted standards of human animal care and approved by Animal Use and Care Committee of National Institute of Child Health and Human Development, National Institutes of Health. *X. laevis* adults were purchased from NASCO (Fort Atkinson, WI). Tadpoles of *X. laevis* were purchased from NASCO or produced and reared in the laboratory.

### LGR5 cloning

Total RNA was extracted from premetamorphic tadpole intestine with TRIZOL reagent (Invitrogen, Carlsbad, CA) and followed by treatment with RNase-free DNase I (Ambion, Austin, TX) to remove any DNA contamination. First-strand cDNA synthesis via reverse transcription (RT) was performed using SuperScript III RT Reaction Kit (Invitrogen, Carlsbad, CA) with RACE (rapid amplification of cDNA ends) primers, random primers, or oligo (deoxythymidine) primers. The primers used were: for 5′ RACE, 5′- GGAACATAGCTGATATGGTTTGCATCC-3′, 5′-GCTCCCTTGGGGATGTATGTCAAGTC -3′; and for 3′ RACE, 5′- CGACTGCAGAAAATAGATCTGCGTC-3′ and 5′-GGCCTTCGTTCCCTAGACTTGGCATGG -3′. The coding region of *X. laevis* LGR5 was cloned by PCR amplification with the primers 5′-ATGGACACCTCCAGGACCAGCTTGTT-3′ (forward) and 5′-GTGACAAGGAACAAATGCCACGG-3′ (reverse). The PCR products were cloned and several independent clones were sequenced to verify the sequences.

### Sequence comparison

The protein sequences of the leucine-rich repeat-containing G protein-coupled receptors were extracted from GeneBank database as the following: human (*Homo sapien,* Hu) luteinizing hormone/choriogonadotropin receptor (Hu-LHR, GenBank accession: NP_000224), follicle stimulating hormone receptor (Hu-FSHR, GenBank accession: NP_000136), thyroid stimulating hormone receptor (Hu-TSHR, GenBank accession: NP_000360), Hu-LGR4 (GenBank accession: NP_060960), Hu-LGR5 (GenBank accession: NP_003658), Hu-LGR6 (GenBank accession: NP_001017403), Hu-LGR7 (GenBank accession: AAG17167), Hu-LGR8 (GenBank accession: NP_570718), chicken (*Gallus gallus*) LGR5 (Ck-LGR5, GenBank accession: XP_425441), gray short-tailed opossum (*Monodelphis domestica*) LGR5 (Md-LGR5, GenBank accession: XP_001370047), and zebra finch (*Taeniopygia guttata*) LGR5 (Tg-LGR5, GenBank accession: XP_002190102).The *X. tropicalis* (Xt) LGR5 nucleotide sequences were identified from the Joint Genome Institute database. The putative *X. laevis* (Xl)-LGR5a, b and Xt-LGR5 protein sequences along with the other LGR protein sequences were analyzed by using CLUSTALW (http://align.genome.jp/).

### RT-PCR

Total RNA was extracted with TRIZOL (Invitrogen) from whole animals at stage 1 (fertilized egg) to stage 66 or isolated intestines, limbs, and tails of *X. laevis* tadpoles at stages 54 (premetamorphosis) to 66 (no tail at this stage, end of metamorphosis) or stage 54 tadpoles after 5 nM T3 treatment for 0 –7 days. The total RNA was treated with RNase-free DNase I (Ambion) to remove any DNA contamination. RT-PCR was performed by using SuperScript III one-step RT-PCR with Platinum*Taq* DNA polymerase (Invitrogen) and 100 ng total RNA. The primers 5′-TACCTTGATCTAAGTATGAACAACAT-3′ (forward) and 5′-GCTCCCTTGGGGATGTATGTCAAGTC-3′ (reverse) were used to determine the expression level of the LGR5 genes by RT-PCR. The expression of ribosomal protein gene (rpl8) [22] was analyzed as an internal control for RNA quantity and quality by including a specific primer pair in the same PCR tubes. The RT-PCR cycle numbers were chosen empirically for each tissue to ensure that the PCR did not reach saturation.

Quantitative real-time RT-PCR (qRT-PCR) with TaqMan probes was carried out to quantify the expression levels of LGR5 mRNA on ABI 7000 (Applied Biosciences, Foster City, CA) as previously described [23]. The primers specific for LGR5 were 5′-GTCTGCATTACAGGCTATGACCTT -3′ (forward), 5′-GCATTTCTTTCCCAGGGAGTAGATT -3′ (reverse), and 5′-(FAM)ATGGAGATGGAGAACTAC(NFQ)-3′ (6-carboxyfluorescein-labeled TaqMan probe). A set of primers/probe specific for ribosomal protein rpl8 [24] was used as the control for RNA input for each sample, and the LGR5 expression level in each sample was normalized to that of rpl8.

### *In situ* hybridization (ISH)

The entire coding region of LGR5 cDNA was subcloned into pBSII-KS^+^. The resulting plasmid was linearized to synthesize sense and antisense probes either with T3 or T7 RNA polymerase, respectively, by using digoxigenin (DIG) RNA Labeling Mix (Roche Applied Science, Indianapolis, IN, USA). Probes were partially hydrolyzed with alkaline treatment (40 mM NaHCO_3_, 60 mM Na_2_CO_3_) to sizes of about 200-bases long. Intestinal fragments were isolated from the anterior part of the small intestine just after the bile duct junction from tadpoles at indicated stages as well as premetamorphic tadpoles after 10 nM T3-treatment and fixed in MEMFA followed by cryo-sectioning. Tissue sections cut at 7 µm were subjected to ISH by using sense or antisense probe as previously described [25]. Some sections were stained by methyl green-pyronin Y to identify the adult epithelial progenitor cells [26]. Photographs were taken by using a digital CCD color camera (DP70, Olympus, Tokyo, Japan) attached to an optical microscope (BX51, Olympus).

## Results

### *Xenopus* LGR5 is highly conserved with its homologs in other vertebrates

To investigate the possible role of LGR5 in the development of the adult intestinal stem cells during metamorphosis in *Xenopus laevis*, we first cloned a partial LGR5 cDNA in *Xenopus laevis* by RT-PCR (data not shown). 5′- and 3′- RACE were performed to determine the sequence of the 5′ and 3′ end followed by PCR amplification of the full length LGR5. Multiple clones were sequenced and the results revealed the existence of two duplicated LGR5 genes (designated as LGR5a and LGR5b, both are highly homologous and only LGR5a was used for most of the sequence comparisons) in the *Xenopus laevis* genome because of its pseudotetraploidy. The coding region of each gene is comprised of 2709 bp and encodes a protein of 902 amino acids (aa) long. The cDNA sequence was used to search the *Xenopus tropicalis* genomic sequence database, revealing a putative *Xenopus tropicalis* LGR5 of 902 aa, which shares about 95.7% identities with the *Xenopus laevis* LGR5 proteins.

**Figure 1:**
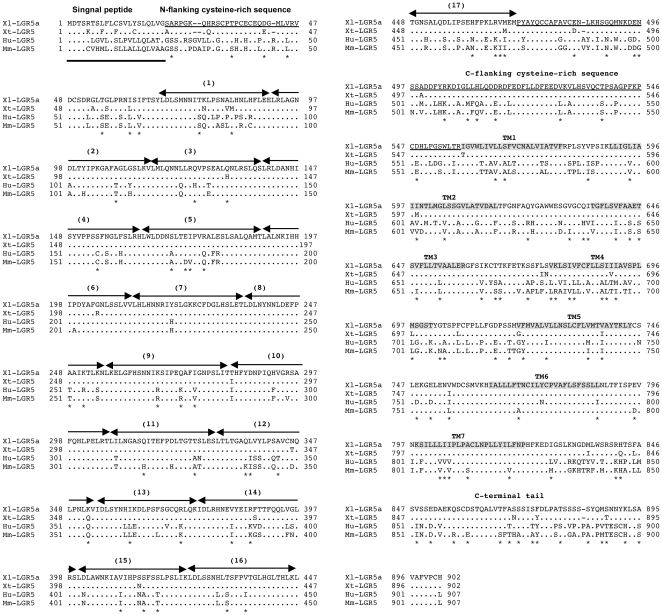
The *X. laevis* (Xl) LGR5 shares a high degree of homology with *X. tropicalis* (Xt), human (Hu) and mouse (Mm) LGR5. There are two duplicated LGR5 genes in *X. laevis* and only one (Xl-LGR5a) was used for comparison. The deduced amino acid (aa) sequences were aligned and gaps were introduced to achieve best alignments among species. Dots indicate the aa are the same as that of Xl-LGR5a and conserved changes among species are marked with stars under the Mm-LGR5 sequences. Number (1) to (17) indicate the highly conserved leucine-rich repeats. The conserved transmembrane domains are indicated with TM1-7. The N-terminal signal peptide, N-, C-flanking cysteine-rich sequences, and C-terminal tail are not well conserved.

**Figure 2:**
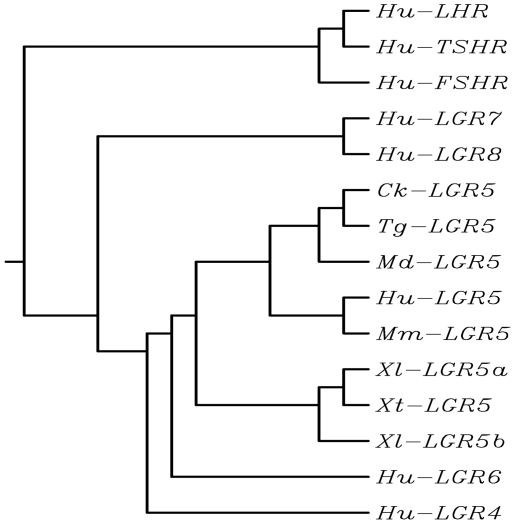
A phylogenetic tree of LGR proteins. Amino acid sequences of indicated LGR proteins were analyzed with Multiple Sequence Alignment by CLUSTALW (http://align.genome.jp/). The proteins form three subgroups: one containing Hu-LHR, Hu-FSHR, and Hu-TSHR; another containing LGR7 and LGR8, whose ligands are small heterodimeric peptides with homology to insulin, including the pregnancy hormone relaxin and insulin-like 3 [33], [34], [35]; and the third containing LGR4, LGR5, and LGR6, including the *X. laevis* LGR5a, b as well as *X. tropicalis* LGR5, and LGR6. Hu, human, Ck, chicken, Tg, *Taeniopygia guttata*, Md: *Monodelphis domestica.*

Pair-wise sequence comparison showed that *Xenopus laevis* LGR5 is 68% and 70% homologous to the mouse and human LGR5 at the nucleotide level, respectively (data not shown). The homology of the putative *Xenopus laevis* LGR5 protein to the mouse and human LGR5 is 71.9% and 73%, respectively (data not shown). Sequence comparison among the *Xenopus laevis, Xenopus tropicalis*, mouse, and human LGR5 proteins suggests that the leucine-rich repeats and the transmembrane region are highly conserved. On the other hand, the N-, C-flanking cysteine-rich sequences and C-terminal tail sequences are more variable (Fig. 1). LGR5 contains 7-transmembrane domains with a large extracellular domain for ligand binding and a short cytoplasmic tail for coupling to G-proteins. This structure is closely related to G protein-coupled receptors with glycoprotein ligands, such as FSHR (LGR1), LHR (LGR2) and TSHR (LGR3). Phylogenetic analysis of the LGR family of proteins demonstrated clearly the putative *X. laevis* LGR5a, b as well as *X. tropicalis* LGR5, cluster closely with the LGR5 in other species but are further away from other family members such as LGR4, LGR6, and TSHR (Fig. 2), indicating that these frog proteins are true homologs of the mammalian LGR5.

### Zygotic LGR5 transcription occurs during late embryogeneis

**Figure 3:**
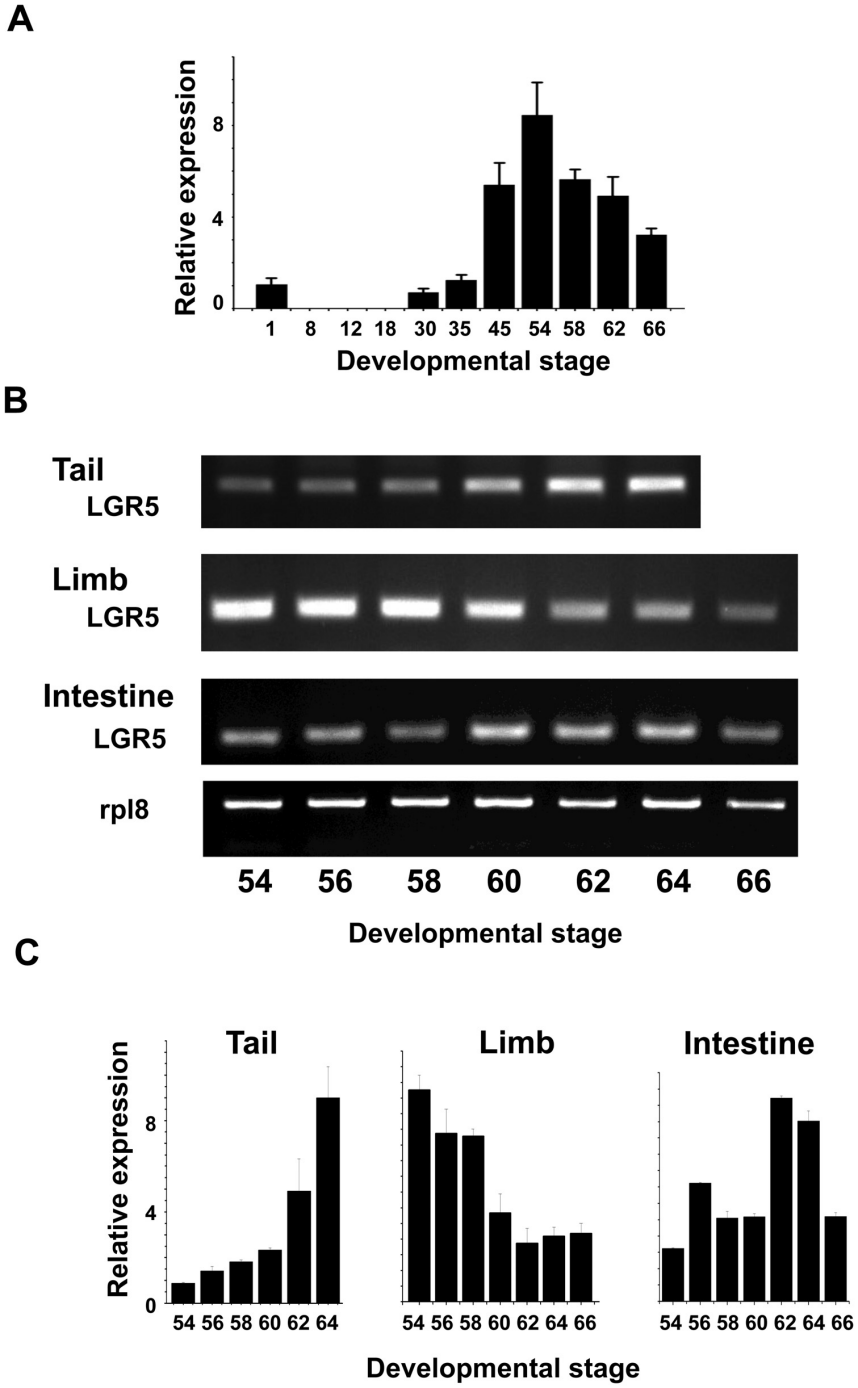
Stage- and tissue-dependent regulation of LGR5 expression during development. (A) Zygotic transcription of LGR5 begins in late embryogenesis during development in *X. laevis*. Total RNA was isolated from whole animals from stage 1 (fertilized egg) to stage 66 (the end of metamorphosis) and analyzed by qRT-PCR. The LGR5 expression shown in (C) was normalized against rpl8 expression determined at the same time. (B, C) Organ-specific temporal regulation of LGR5 during natural metamorphosis in *X. laevis*. Total RNA was isolated from tail, limb, and intestine of tadpoles at the indicated developmental stages and used for regular RT-PCR (B) or qRT-PCR (C) analysis of the LGR5 expression. In each RT-PCR, a primer set for the ribosomal gene rpl8 was also included in the same PCR reaction as an internal control, although the rpl8 result was shown only for the intestine in (B). The LGR5 expression shown in (C) was normalized against rpl8 expression determined at the same time.

To investigate the expression of LGR5 during development, we isolated RNA from whole animals at stage 1 (fertilized egg) to stage 66 (the end of metamorphosis when the tail is no longer present) and subjected it to qRT-PCR analysis. LGR5 was found to be a maternal gene with significant expression in fertilized egg (Fig. 3A). Its mRNA then became non-detectable until zygotic transcription began at stage 30, right before hatching around stage 35. By tadpole feeding stage 44/45, its mRNA reached high levels and remained highly expressed till the end of metamorphosis. Thus, LGR5 likely plays a role for tadpole growth during premetamorphosis as well as for metamorphic transformations.

### LGR5 expression correlates with organ-specific metamorphosis

Different organs undergo metamorphosis at different developmental stages. It has been well-established that in *X. laevis*, the tail undergoes complete resorption through apoptosis near the end of metamorphosis, mainly between stages 60 and 66, while the limbs undergo *de novo* development very early, with its growth and morphogenesis taking place around stages 54-58 [27]. Most other organs are remodeled during metamorphosis. One of the best studied example is the intestine, which undergoes extensive remodeling involving both cell death and cell proliferation followed by differentiation [5]. To investigate the possible involvement of LGR5 in *Xenopus laevis* metamorphosis, we analyzed the temporal regulation of the two duplicated LGR5 genes in the limb, tail, and intestine at different stages by RT-PCR and qRT-PCR (as the two duplicated genes are highly homologous, we analyzed the expression of both genes together). Our results showed that LGR5 expression was up-regulated dramatically after stage 60 in the tail when tail resorption occurs (Fig. 3B, C). In the limb, LGR5 mRNA was down-regulated after stage 58 (Fig. 3B, C). Interestingly, the high levels of LGR5 expression between stages 54 and 58 correlated with limb growth and morphogenesis. Likewise, in the intestine, its expression peaked around stages 62–64 when rapid proliferation of adult epithelial cells occurred (Fig. 3B, C) [5]. These results suggest that LGR5 regulation correlates with organ–specific changes during frog metamorphosis.

### T3 induces LGR5 expression in different organs

**Figure 4:**
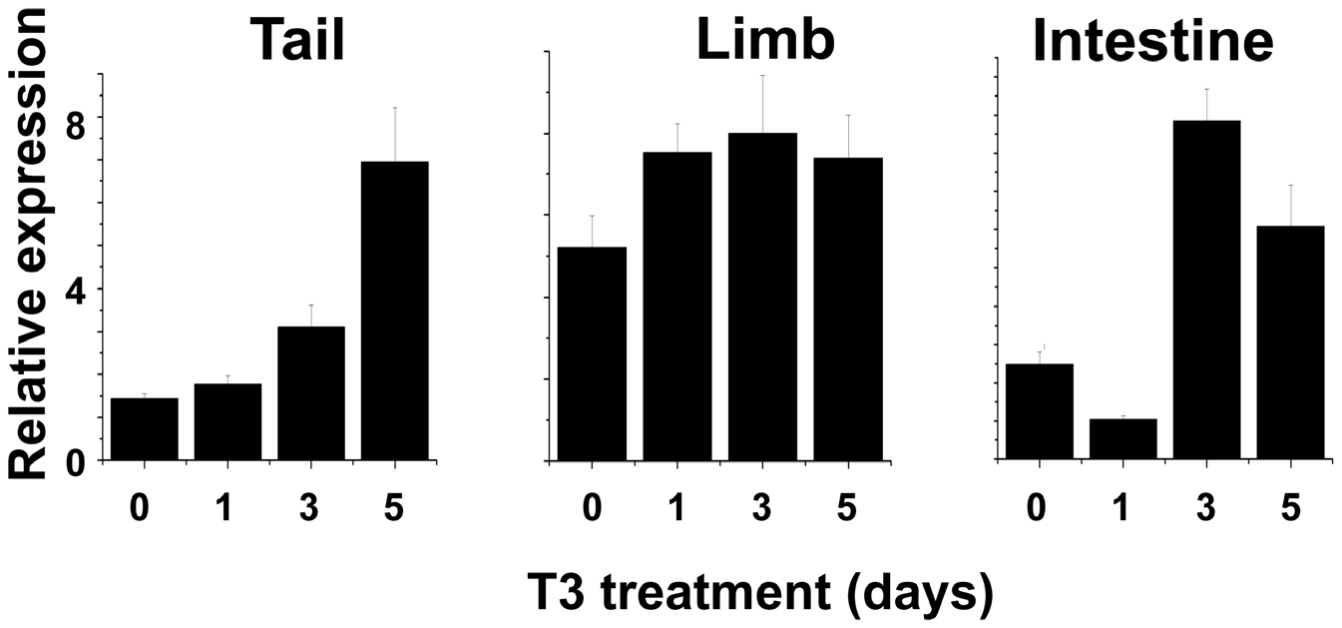
T3 up-regulates LGR5 expression in premetamorphic tadpoles. Tadpoles at stage 54 were exposed to 5 nM T3 for 0–5 days before tissue isolation, RNA extraction, and qRT-PCR analyses as in Fig. 3C.

As T3 controls the changes in all organs during metamorphosis, the correlation of LGR5 expression with metamorphosis in different organs suggests that LGR5 expression is directly or indirectly regulated by T3. To test this possibility, we treated premetamorphic tadpoles at stage 54 with 5 nM T3, about the peak plasma level during natural metamorphosis [28], and isolated total RNA from the limb, tail, and intestine. As shown in Fig. 4, qRT-PCR analysis showed that in the tail and intestine, LGR5 expression was up-regulated after 3-5 days of T3 treatment, consistent with the expression pattern during natural metamorphosis. In the limb, T3 treatment slightly upregulated LGR5 expression (Fig. 4). This likely reflected the fact that by stage 54, LGR5 expression was already very high in the limb (Fig. 3B, C) even though endogenous T3 level was still quite low [28]. During natural metamorphosis, LGR5 expression was eventually down-regulated near the end of metamorphosis (Fig. 3B, C). Expectedly, such down-regulation was not observed during the T3-treatment due to the relative short treatment period. Thus, T3 regulates the expression of LGR5 during metamorphosis, although the slow induction of the gene in the tail and intestine suggests that LGR5 is not directly regulated by T3 at the transcription level.

### LGR5 is expressed in the proliferating progenitor/stem cells of the intestine

The development of the adult intestine during metamorphosis involves the degeneration of the larval epithelium and *de novo* development of the adult one [5]. Recent studies have shown that the adult progenitor/stem cells of intestinal epithelium develop from the larval epithelium, likely through dedifferentiation around stages 60 [5], [11], [12], [15]. While the underlying mechanism is unclear, the proliferating adult intestinal epithelial cells can be easily identified as multi-cell islets or cell nests around stage 60 [4], [5]. The high levels of LGR5 expression at stages 62–64 in the intestine suggest that LGR5 is expressed in the proliferating adult epithelial cells. To investigate this possibility, *in situ* hybridization (ISH) was carried out on intestinal sections from tadpoles at different stages.

**Figure 5:**
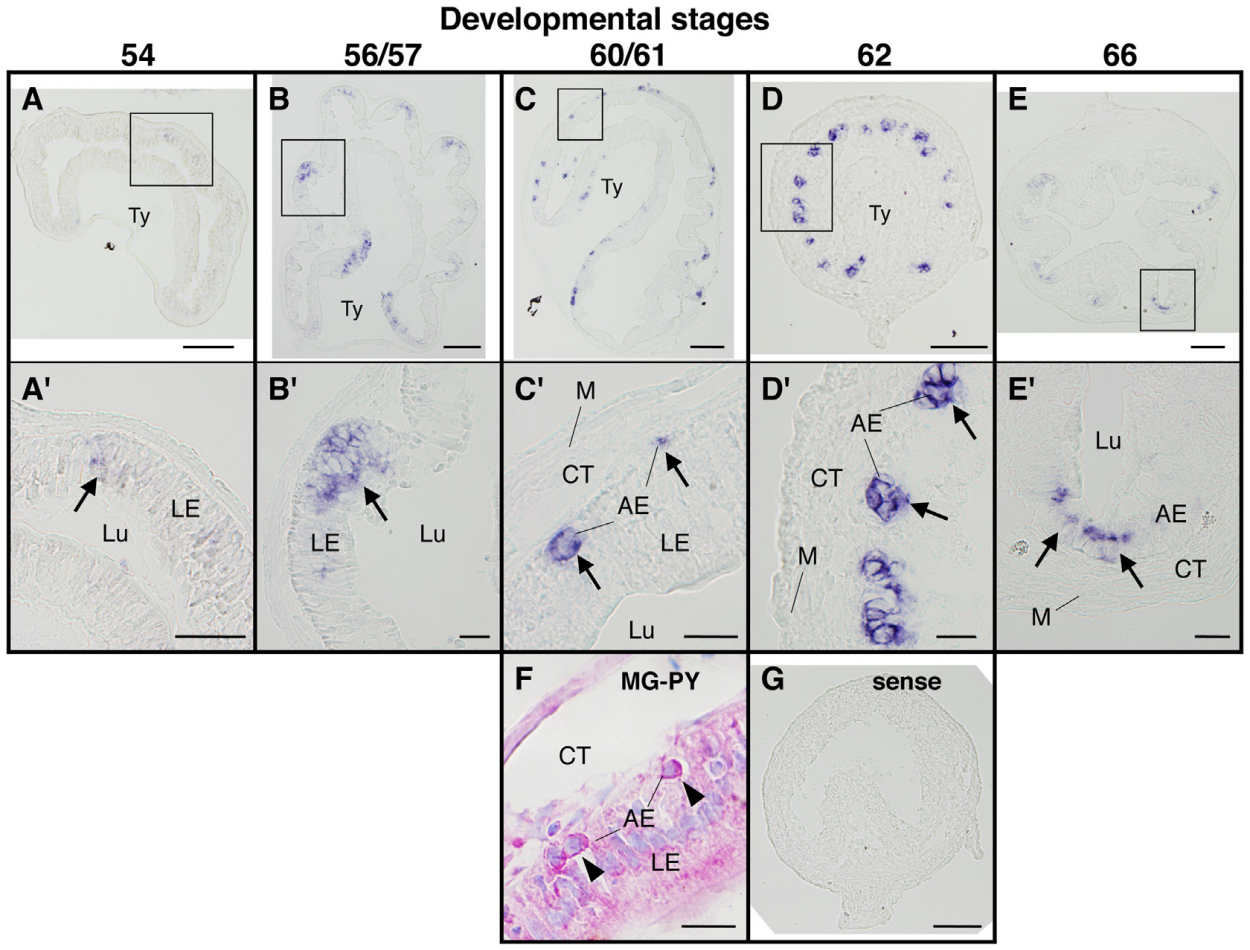
Spatiotemporal expression of LGR5 mRNA in the small intestine during natural metamorphosis. Cross sections of the intestine at premetamorphic stage 54 (A, A′), prometamorphic stage 56/57 (B, B′), metamorphic climax stages 61 (C, C′, F) and 62 (D, D′, G), and the end of metamorphosis (stage 66) (E, E′) were hybridized with LGR5 antisense (A–E′) or sense probe (G). To compare the localization of LGR5 mRNA (C, C′) with that of adult epithelial progenitor cells, the serial sections at stage 60/61 were stained with methyl green-pyronin Y (MG-PY) (F). Arrows indicate the cells expressing LGR5 (A′–E′), while arrowheads indicate adult epithelial progenitor cells (F). Higher magnification of boxed areas in (A)–(E) are shown in (A′)–(E′). Sense probe did not produce any signal (G). Note that at metamorphic climax stage 61, LGR5 mRNA was localized in the islets between the larval epithelial cells and the connective tissue (C, C′). These islet cells were identified as the adult epithelial progenitor cells strongly stained red with pyronin Y (F) [29]. AE: adult epithelial cell including progenitor/stem cell, CT: connective tissue, LE: larval epithelial cell, Lu: lumen, M: muscle layer, Ty: typhlosole. Scale bars are 100 µm (A–E, G) and 20 µm (A′–E′, F), respectively.

The ISH results showed that the expression of LGR5 mRNA was epithelial cell-specific in the intestine throughout metamorphosis (Fig. 5). In agreement with the RT-PCR analysis shown in Fig. 3, the level of LGR5 was very low and LGR5-positive cells were few at the premetamorphic stage 54 (Fig. 5A). At this stage, cells weakly expressing LGR5 mRNA were rarely and randomly distributed in the epithelium (Fig. 5A, A′). During prometamorphosis, these cells increased in number (Fig. 5B, B′). It is important to note that until this stage, the intestinal epithelium consists of essentially only the differentiated larval cells. At the climax stage 60/61, stem/progenitor cells of the adult epithelium become detectable as islets that consist of undifferentiated cells strongly stained red with pyronin Y [29] between the larval epithelial cells, which are undergoing apoptosis [5], and the connective tissue (Fig. 5F). The level of LGR5 mRNA became higher and specifically localized in these islets (Fig. 5C, C′). Concomitantly with the increase of the adult epithelial cells in number due to active proliferation at stage 62, the number of LGR5-positive cells increased (Fig. 5D, D′). At the end of metamorphosis (stage 66), when the intestinal remodeling is completed, LGR5 mRNA became localized in the trough region of the intestinal folds (Fig. 5E, E′), where the adult epithelial stem cells are localized [4], [26].

**Figure 6:**
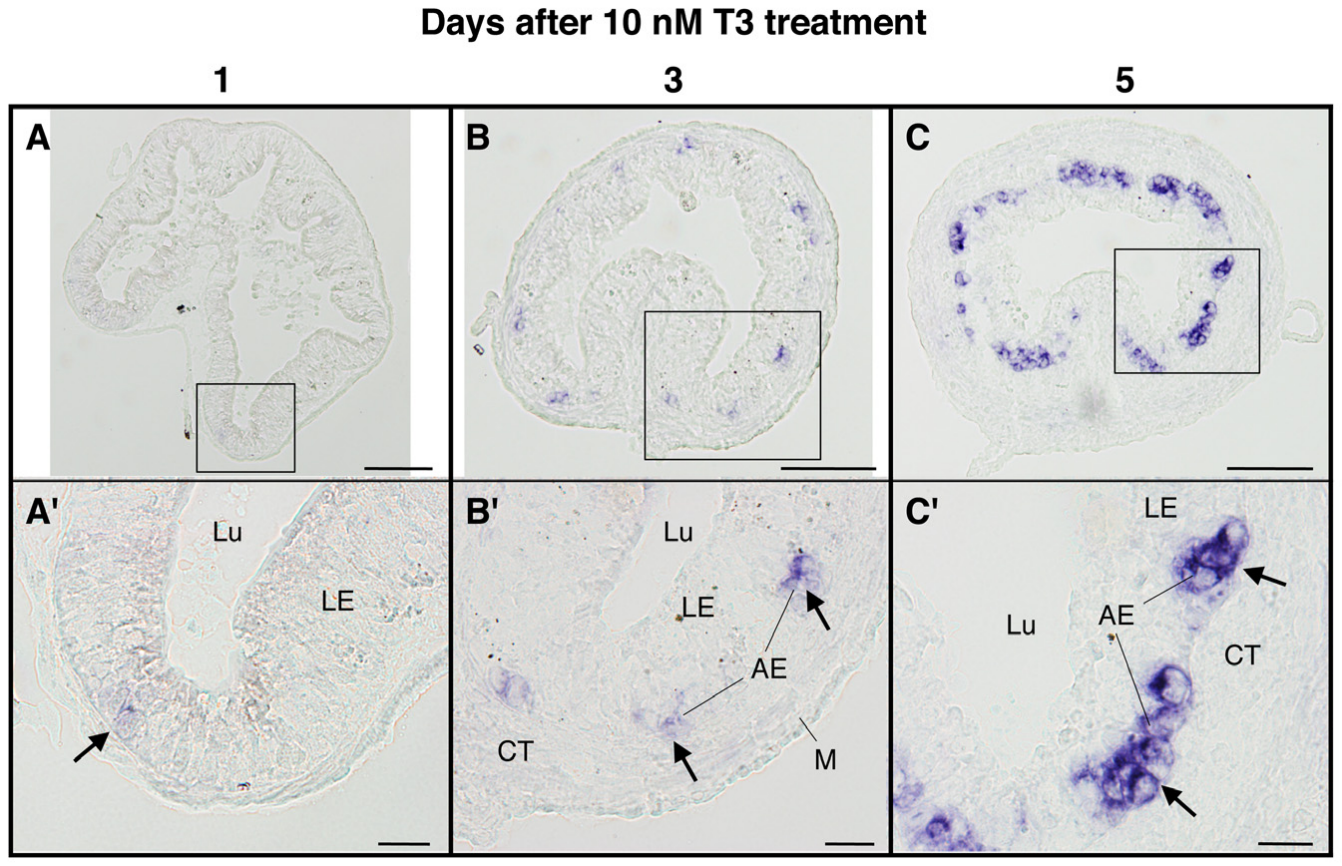
T3-dependent expression of LGR5 mRNA in the small intestine. Cross sections of the intestine from premetamorphic tadpoles (stage 54) treated with 10 nM T3 for 1 (A, A′), 3 (B, B′) and 5 days (C, C′) were hybridized with LGR5 antisense probe. AE: adult epithelial cell including the progenitor/stem cell, CT: connective tissue, LE: larval epithelial cell, Lu: lumen, M: muscle layer. Scale bars are 100 µm (A–C) and 20 µm (A′–C′), respectively.

Given the T3-dependence of intestinal metamorphosis and the up-regulation of LGR5 by T3, we next examined the expression patterns of LGR5 mRNA after T3 treatment of premetamorphic tadpoles at stage 54 (Fig. 6). For this experiment, tadpoles were treated with 10 nM T3 to induce precocious metamorphosis. After 1 day of T3 treatment, there was little change in the expression of LGR5 (Fig. 6A, A′). Its expression was up-regulated after 3 and 5 days of T3 treatment (Fig. 6B, B′, C, C′). The expression patterns were essentially the same as those during natural metamorphosis (compare Fig 6 with Fig. 5). That is, during both natural and T3-induced metamorphosis, the expression of LGR5 mRNA was restricted to the newly developed, proliferating adult cells but not the larval epithelial cells undergoing apoptosis, suggesting that this gene plays a role in development of the adult epithelial progenitor/stem cells and may also serve as a good molecular marker for them.

## Discussion

LGR5 was originally identified from human placenta as a homolog of glycoprotein hormone receptors such as receptors for LH, FSH and TSH [19] and was subsequently shown to be a target gene of Wnt signaling [17], [30]. In human, LGR5 is strongly expressed in the muscle, placenta and spinal cord and weakly expressed in the brain, colon, small intestine, stomach, bone marrow and adrenal [19], suggesting that the role of LGR5 is tissue/organ dependent and that transcriptional control of LGR5 gene varies among the tissues/organs. Indeed, our results here show that during natural and T3-induced metamorphosis, LGR5 may have distinct functions in different organs, involving in stem cell function in the intestine and possibly limb but likely playing a different role in the tail. Furthermore, such roles appear to be conserved in a highly related frog species, *Xenopus tropicalis*, as similar developmental and T3-dependent expression profiles were also observed in this species (data not shown).

One of the most interesting findings on LGR5 is its specific expression in the stem cells of mouse intestine [17]. More importantly, a single LGR5-positive stem cell can generate the crypt-villus structure *in vitro* and that LGR5 deficiency causes precocious Paneth cell differentiation in the fetal intestine [16], [17], [18]. These results suggest that LGR5-positive cells in the intestine are true intestinal stem cells and that LGR5 regulates intestinal stem cell differentiation in mouse.

Here we have investigated the potential role of LGR5 during the development of adult intestinal epithelium during postembryonic development in frogs. Intestinal metamorphosis is an excellent model to study the development of adult organ-specific stem cells. There are no identifiable adult stem cells in the tadpole intestine and all larval epithelial cells appears to be differentiated although at least some of them are mitotically active [4], [5], [26]. During metamorphosis, T3 induces the larval cells to die and for unknown reason, some cells from the larval epithelium escape this apoptosis and dedifferentiate to become the progenitor/stem cells for the adult epithelium. The up-regulation of LGR5 during intestinal development correlates well with the appearance of adult epithelial progenitor cells. LGR5 expression was also detected, although at low levels, in the premetamorphic intestine at as early as stage 44/45 when feeding just begins and its expression level remained fairly constant till stage 54 (data not shown). As metamorphosis proceeds, more cells with higher levels of LGR5 expression appear. Since larval epithelial cells are mitotically active [4], [5], [26], it is tempting to speculate that the few larval epithelial cells with detectable LGR5 expression are destined to dedifferentiate into the adult progenitor/stem cells. Consistently, as metamorphosis proceeds, LGR5 was specifically detected in the proliferating cells. In addition, at the end of metamorphosis, LGR5 was detected in the trough region of the intestinal folds where the epithelial stem cells reside in the adult frog intestine, similar to that in the mouse intestine [17], [18]. Therefore, our results suggest that the up-regulation of LGR5 expression in larval intestinal epithelial cells marks those cells that eventually dedifferentiate into the adult epithelial stem/progenitor cells.

The spatiotemporal expression pattern also suggests that LGR5 play a role in the development/maintenance of the adult stem cells in the intestinal epithelium. In this regard, it will be important to identify the ligand(s) for LGR5 during metamorphosis. Interestingly, the insect cuticle-hardening hormone Bursicon consists two proteins, encoded by the genes burs and pburs (partner of burs), and the pburs/burs heterodimer binds with a high affinity to *Drosophila* LGR2 [31], the fly ortholog of vertebrate LGR4, LGR5, and LGR6. These proteins are similar to vertebrate BMP antagonists, such as gremlin and Cerberus. It is possible that BMP antagonists function as LGR5 ligands to affect the development of intestinal stem cells. In fact, one of the BMPs, BMP4, is upregulated in the connective tissue during intestinal metamorphosis [32], raising the possibility that spatiotemporal regulation of BMP activity is important for the development of the adult intestine.

The slow induction of the expression of LGR5 by T3 suggests that LGR5 is not directly regulated by T3 via its nuclear receptor at the transcription level. In mammals, LGR5 is a known Wnt target gene [30]. It is likely that Wnt 5a, which is up-regulated during intestinal remodeling [24], may be responsible for the up-regulation of LGR5 in *X. laevis* intestine, just like in mouse. Future studies to decipher how LGR5 is upregulated in the adult intestinal progenitor/stem cells may provide mechanistic insight on how these cells are developed from the larval epithelium during metamorphosis.
